# EGF-supplemented decellularized tracheal matrix hydrogels promote tracheal defect repair

**DOI:** 10.1186/s13019-026-04297-1

**Published:** 2026-06-12

**Authors:** Qingxu Hao, Wenyu Song, Peng Zuo, Liangbin Pan

**Affiliations:** 1 Department of Thoracic Surgery, The First Affiliated Hospital of Soochow University,Medical College of Soochow University, Soochow University, Suzhou, 215000 Jiangsu China; 2 Institute of Thoracic Surgery, The First Affiliated Hospital of Soochow University, Suzhou, China

**Keywords:** Tissue engineering trachea, Decellular matrix (DCM), Polyethylene glycol diacrylate (PEGDA), Epidermal growth factor (EGF), Tracheal epithelium

## Abstract

**Background:**

Tracheal defect repair is a major clinical challenge in thoracic surgery, with long-segment defects having a low clinical repair rate. Autologous cartilage transplantation is limited by high postoperative stenosis and insufficient autologous tissue, while traditional artificial tracheal materials have poor biocompatibility and no epithelial regeneration-inducing capacity, leading to poor long-term efficacy. This study aimed to fabricate a biomimetic composite hydrogel and evaluate its physicochemical properties and tracheal repair efficacy, providing a new tissue-engineered strategy for clinical tracheal defect management.

**Methods:**

A PEGDA/DCM/EGF composite hydrogel was prepared via photopolymerization. Its physicochemical properties and EGF release pattern were characterized. In vitro experiments on rat tracheal epithelial cells assessed cell viability, proliferation and migration. A tracheal defect model was established in SD rats, with the hydrogel implanted; gross observation, HE staining and immunohistochemistry were performed at 2 weeks post-implantation to evaluate repair efficacy, with statistical methods used for intergroup comparisons.

**Results:**

The PEGDA/DCM hydrogel had optimized microstructure and mechanical properties matching native trachea, maintained structural stability under physiological conditions, and achieved sustained EGF release without burst effect. The PEGDA/DCM/EGF hydrogel significantly promoted the viability, proliferation and migration of rat tracheal epithelial cells in vitro. In vivo, implanted SD rats had intact tracheal architecture and unobstructed lumens at 2 weeks; HE staining and immunohistochemistry confirmed continuous epithelial layer formation and successful epithelialization at the defect site.

**Conclusions:**

The PEGDA/DCM/EGF composite hydrogel has favorable physicochemical properties, excellent biocompatibility and effective tracheal repair efficacy, with DCM optimizing hydrogel performance and sustained EGF release accelerating epithelial regeneration. This study provides a promising tissue-engineered strategy for tracheal defect repair, with significant clinical translation potential for thoracic surgery clinical practice.

## Introduction

The trachea not only serves as the key hub connecting the larynx and the bronchi in the respiratory system, but also plays a core role in gas conduction, defense and protection through mucociliary clearance and mucus secretion. At the same time, it precisely regulates airway resistance with the help of the contraction and relaxation of tracheal smooth muscle to ensure smooth and efficient breathing process [1, 2]. However, congenital tracheal malformation, radical resection of malignant tumors, severe trauma, intractable infection and other clinical factors may lead to varying degrees of tracheal defects. If this kind of defect is not repaired in time and effectively, it is easy to cause airway obstruction, repeated infection, respiratory failure and other serious complications, which not only seriously reduces the quality of life of patients, but also directly threatens the safety of life [3, 4].

At present, the commonly used clinical methods for tracheal defect repair have inherent limitations that are difficult to break through. While autologous tissue transplantation (such as thyroid cartilage and costal cartilage transplantation) can avoid the risk of immune rejection, autologous tissue sources are limited and cannot meet the repair needs of large-segment tracheal defects. Furthermore, the surgical trauma is significant and may lead to complications such as autologous donor site pain and deformity [5]. Meanwhile, the anatomical structure and mechanical properties of the transplanted tissue cannot fully match those of the native trachea, leading to a high incidence of postoperative airway stenosis and collapse [6]. Although allogeneic tracheal transplantation can provide a relatively sufficient source of donors, it is difficult to effectively control the strong immune rejection caused by it. Long-term use of immunosuppressants will also lead to a series of side effects such as decreased immunity, liver and kidney function damage [7]. Artificial trachea is mostly made of metal, synthetic polymer and other materials. Although it can temporarily maintain airway patency, it has poor biocompatibility, lack of biological activity. After long-term implantation, it is easy to cause infection, granulation tissue proliferation, airway stenosis and other problems, which seriously affect the long-term effectiveness of repair [8].

With the rapid development of biomedical engineering technology, tissue engineering technology with multivariate system as the core provides a new solution for tracheal defect repair [9, 10]. An ideal tissue-engineered tracheal scaffold must meet multiple key characteristics simultaneously: it should possess good biocompatibility to avoid host immune rejection and toxic damage; have appropriate mechanical properties that match the dynamic mechanical environment of the trachea during breathing and swallowing, specifically providing sufficient structural support to maintain lumen patency while having certain elasticity and toughness to adapt to deformation during physiological activities; feature a biomimetic structure that simulates the extracellular matrix (ECM) microenvironment of the natural trachea, thereby offering suitable spatial conditions for cell adhesion, proliferation and differentiation; and additionally have efficient drug or growth factor delivery capacity to provide continuous biological signals for tissue regeneration [11–14].

Among the many scaffold materials, hydrogels can provide a moist and breathable biomimetic microenvironment for cell growth due to their high water content (usually above 90%) and three-dimensional network structure similar to the natural extracellular matrix [15, 16]. Their physical and chemical properties can be flexibly regulated by the ratio of raw materials and cross-linking, and they also have good biocompatibility and drug loading capacity [17, 18]. It has become a research hotspot in the field of tissue engineering. Among them, polyethylene glycol diacrylate (PEGDA) is a widely used hydrogel matrix material, which has excellent biocompatibility, low immunogenicity and good machinability. It can quickly prepare structurally stable hydrogels through ultraviolet light cross-linking and other methods. Moreover, its pore size, mechanical strength and other physical and chemical properties can be precisely regulated by adjusting molecular weight, concentration and other parameters [19–21]. However, the simple PEGDA hydrogel lacks bioactive sites and is difficult to effectively support cell adhesion and proliferation, which cannot meet the biological needs of tissue regeneration [22].

Decellularized matrix (DCM) is a natural extracellular matrix scaffold that is retained after the removal of cellular components and immunogenic substances from natural tissues through a combination of physical, chemical, and biological methods. DCM completely retains a variety of bioactive components such as collagen, elastin, glycoaminoglycan, and fibronectin, which can provide abundant adhesion sites for cells [23]. At the same time, its natural three-dimensional structure simulates the extracellular matrix microenvironment, effectively regulates cell adhesion, proliferation, differentiation and migration behavior, and significantly enhances the biological activity of the scaffold [24]. Epidermal growth factor (EGF), as an important pro-cell growth factor, can specifically bind to the EGF receptor on the surface of epithelial cells, activate the downstream PI3K-Akt, MAPK/ERK signaling pathways, effectively promote the proliferation and migration of epithelial cells, and accelerate the epithelization and repair of damaged tissues [25–27]. It plays an irreplaceable key role in the repair process of skin, mucosa, airway and other tissue injuries.

Based on the above research background, we constructed a PEGDA/DCM/EGF composite hydrogel scaffold by combining polyethylene glycol diacrylate (PEGDA), tracheal decellularized matrix (DCM), and epidermal growth factor (EGF). We hypothesized that the composite hydrogel could achieve functional synergy among the three components: the PEGDA backbone provided stable mechanical support and a controllable 3D network structure; the tracheal decellularized matrix (DCM) supplied bioactive components to establish a biomimetic extracellular matrix microenvironment; and the hydrogel network enabled sustained EGF release to deliver long-term biological signals for tissue regeneration. The synergistic effect of these three components is expected to significantly enhance tracheal defect repair.

**Scheme 1:**
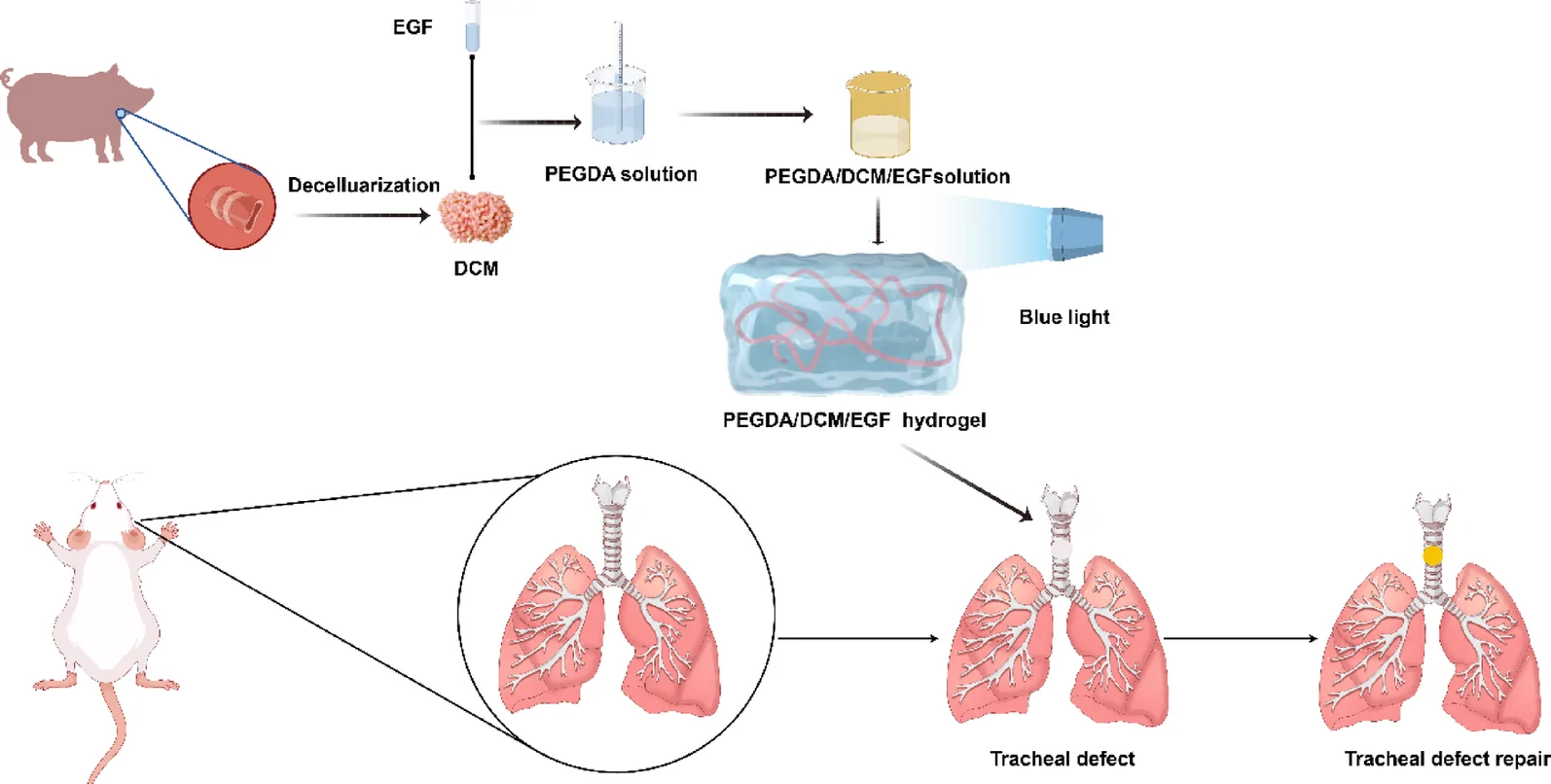
Schematic diagram illustrating the fabrication of PEGDA/DCM/EGF hydrogels and their applications in tracheal defect repair

To test this hypothesis, we systematically characterized the physicochemical properties of the composite hydrogel, including its microstructure, mechanical properties, viscoelasticity, swelling behavior, and EGF release profile. We also evaluated its in vitro biocompatibility using live/dead staining, CCK-8 assay, and scratch wound healing test. Furthermore, we assessed its in vivo tracheal repair efficacy using a rat tracheal defect model. This study aimed to verify the feasibility and superiority of the composite hydrogel as a tissue engineered tracheal scaffold, and to provide a reliable experimental foundation for its future clinical translation Scheme 1.

## Materials and methods

### Experimental materials

Polyethylene glycol dimethacrylate (PEGDA, molecular weight 5000), photoinitiator (Irgacure 2959), and sodium dodecyl sulfate (SDS) were purchased from Sigma-Aldrich in the United States; rat epidermal growth factor (EGF) was purchased from PeproTech in the United States; fetal bovine serum (FBS), DMEM/F12 medium, and penicillin - streptomycin double antiserum were purchased from Gibco in the United States; CCK-8 kit, live/dead staining kit, and HE staining kit were purchased from Bio-Rad in China; Cytokeratin 19 (CK19) primary antibody and FITC-labeled secondary antibody were purchased from Abcam in the United Kingdom; rat EGF enzyme-linked immunosorbent assay (ELISA) kit was purchased from Shanghai Linkong Biotechnology Co., Ltd.; male SD rats (weight 200–250 g) were purchased from Beijing Vantonglihua Laboratory Animal Technology Co., Ltd.; rat tracheal epithelial cells (RTEpiC) were purchased from Shanghai Zhongqiu Xinzhou Biotechnology Co., Ltd.

### Preparation of tracheal decellularized matrix (DCM)

Obtain fresh pig trachea, remove non-cartilage tissue, trim the tracheal cartilage and repeatedly wash with phosphate-buffered saline (PBS). Immerse the cartilage tissue in 10 mM Tris-HCl hypotonic buffer (pH 8.0) for constant temperature treatment for 12 h, then transfer to Tris buffer salt water containing 1% SDS for continuous stirring for 2 h. Centrifuge at 2000 rpm for 5 min to collect the tissue sediment, and place the sediment in a system containing DNase for 12 h at 37 ℃ constant temperature incubation to degrade residual nucleic acids. After repeated washing with deionized water until the washing solution is clear, freeze-dry the tissue, and then crush it at -198 ℃ to obtain tracheal decellularized matrix powder.

### Preparation of PEGDA/DCM/EGF composite hydrogel

Dissolve PEGDA in PBS to prepare a 15% (w/v) PEGDA solution; add DCM powder to the solution to a final concentration of 1% (w/v), ultrasonicate for 30 min; add photoinitiator Irgacure 2959 to a final concentration of 0.5% (w/v), stir evenly, and incubate in the dark for 30 min to obtain PEGDA/DCM pre-polymer solution. Dissolve EGF powder in PBS to prepare a 50 µg/mL stock solution, add it to the PEGDA/DCM pre-polymer solution at a final concentration of 1 µg/mL, gently stir evenly (avoid generating bubbles), and prepare PEGDA/DCM/EGF pre-polymer solution. Inject the PEGDA, PEGDA/DCM, and PEGDA/DCM/EGF pre-polymer solutions into custom molds (diameter 8 mm, height 2 mm), place them in the UV crosslinking instrument, irradiate at a light intensity of 15 mW/cm² for 60 s, and obtain the corresponding hydrogels.

### Physicochemical performance characterization of hydrogels

#### Scanning electron microscopy (SEM) observation

Perform gold spraying treatment on the freeze-dried hydrogel samples (spray time 60 s, current 15 mA), and use the Hitachi SU8010 scanning electron microscope from Japan to observe the microscopic morphology of the hydrogels under an accelerating voltage of 5 kV.

#### Mechanical performance test

The hydrogel was prepared as a cylindrical sample with a diameter of 10 mm and a height of 5 mm. The compression test was conducted using the Instron 5967 universal material testing machine from the United States, with a test speed of 1 mm/min. The stress-strain curve and the maximum compressive stress were recorded.

#### Rheological analysis

The hydrogel was characterized at 37 °C using an Anton Paar MCR 302 rheometer. All tests were performed with parallel-plate geometry (20 mm diameter, 1 mm gap). The storage modulus (G′) and loss modulus (G″) of the hydrogel were determined via three rheological measurements: time sweep (fixed frequency 1 Hz, duration 0–1500 s), frequency sweep (0.1–10 Hz, strain 1%, test duration 30 min), and strain sweep (fixed frequency 1 Hz, strain range 0–20%, test duration 30 min).

#### Swelling experiment

The initial weight of the hydrogel (W0) was accurately weighed. It was then immersed in 37 °C PBS. Samples were taken at 1, 3, 5, 7, 12, 24, and 36 h and weighed (Wt) after drying the surface with filter paper. The swelling ratio (SR) was calculated using the formula (1):

1 $$\mathrm{SR} = \text{(Wt - W0)/W}0 \times 100\% $$

#### EGF release curve measurement

A PEGDA/DCM/EGF hydrogel sample containing 100 ng EGF was immersed in 1 mL PBS and incubated in a 37 °C, 50 r/min constant temperature shaker. 0.5 mL of the supernatant was collected at preset time points, and the same volume of fresh PBS was added. The concentration of EGF in the supernatant was determined using an ELISA kit, and the cumulative release rate was calculated and the release curve was plotted. The Release Rate was calculated using the formula (2):

2 $$\begin{aligned}&\text{Release Rate} (\%)=\\&\frac{{C}_{n}\times\:{V}_{\mathrm{total}}+{\sum\:}_{i=1}^{n-1}{C}_{i}\times\:{V}_{\mathrm{sample}}}{{M}_{\mathrm{loaded}}}\times\:100\%\end{aligned}$$

(C_n_: EGF concentration at nth time point, ng/mL; V_total_: total release medium volume, 1 mL; C_i_: EGF concentration at ith time point, ng/mL; V_sample_: sampling volume per time point, 0.5 mL; M_loaded_: initial loaded EGF amount, 100 ng)

### In vitro biocompatibility evaluation

#### Preparation of hydrogel extraction solution

The sterilized hydrogel was immersed in 0.1 g/mL DMEM/F12 medium at 37 °C, and extracted for 24 h in a 37 °C, 50 r/min constant temperature shaker. The extraction solution was filtered through a 0.22 μm sterile filter membrane, sterilized, and stored at 4 °C. It was restored to 37 °C before use.

#### Cell culture

Rat tracheal epithelial cells (RTEpiC) were cultured in DMEM/F12 medium containing 10% FBS and 1% penicillin - streptomycin double antiserum. The cells were cultured in a 37 °C, 5% CO₂ constant temperature incubator, with cell replacement every 2–3 days. When the cell confluence reached 80%-90%, they were passaged. The cells were inoculated into 96-well plates (1 × 10⁴ cells per well), 24-well plates (5 × 10⁴ cells per well), and 6-well plates (2 × 10⁵ cells per well). After 24 h of culture, the control group was added with fresh DMEM/F12 medium, and the experimental group was added with the hydrogel extraction solution (diluted with fresh medium at a 1:1 ratio). Each group had 3 parallel wells.

#### Viability and death staining

After 3 days of co-culture with the extraction solution, the culture medium was aspirated, and PBS was washed 3 times. The staining solution containing 2 µmol/L Calcein-AM and 4 µmol/L PI was prepared according to the instructions of the viability and death staining kit. The staining solution was added to the wells to cover the cells, and incubated at 37 °C, 5% CO₂, and in the dark for 15 min. The staining solution was aspirated, and PBS was washed 3 times. The cells were observed and photographed using a BX53 fluorescence microscope from Olympus.

#### CCK-8 experiment

After co-culturing the cells with the extract for 3 days, the culture medium was removed and the cells were washed 3 times with PBS. 10% CCK-8 reagent in serum-free DMEM/F12 medium was added to each well, and the cells were incubated at 37 °C with 5% CO₂ for 2 h. The absorbance values (OD values) of each well were measured at 450 nm wavelength using the Swiss Tecan Company’s Infinite M200 Pro microplate reader. The blank well was used to zero the reading, and the results indirectly reflected the cell proliferation activity.

#### Scratch wound healing experiment

Cells were seeded in 6-well plates and cultured until full confluence. Three parallel scratches were made perpendicular to the bottom of the wells using a 200 µL sterile pipette tip. Subsequently, the cells were washed three times with PBS to remove floating cells, and the initial scratch width was recorded (W_0_). The control group was supplemented with DMEM/F12 medium containing 1% FBS, while the experimental group was added with a mixed medium consisting of 1% FBS and gel extract (1:1 dilution). Images of the same visual fields were captured using an Olympus CKX41 inverted microscope (Japan) at 0, 12, 24, 48, and 72 h post-scratching. The scratch width at each time point was measured by ImageJ software (W_t_). The scratch healing rate (HR) was calculated using formula (1):

1 $$\mathrm{HR}=\mathrm{(W0-Wt)/W}0 \times 100\%$$

### In vivo tracheal repair efficacy evaluation

#### Establishment of rat tracheal defect model

The animal experimental protocol was approved by the Ethics Committee of Soochow University (SUDA20251015A01). SD rats were fasted for 12 h and dehydrated for 4 h before being anesthetized by intraperitoneal injection of 10% chloral hydrate (3 mL/kg). They were then placed on a surgical table in a supine position, with hair removed from the neck and the area disinfected and covered with a sterile gauze. The skin along the midline of the neck was incised, and the tissues were separated layer by layer to expose the trachea. A 2 mm long tracheal segment was cut between the 3rd and 4th cartilage rings to establish the defect model. The hydrogel of different experimental groups was trimmed to match the defect shape and implanted into the defect site, then sutured and closed layer by layer. Penicillin (200,000 U/kg) was injected intramuscularly every day for 3 days after the surgery to prevent infection.

#### Sample collection and histological analysis

Two weeks after the surgery, the rats were anesthetized by intraperitoneal injection of 10% chloral hydrate, and the tracheal tissue containing the repaired area was removed from the thoracic cavity and fixed in 4% paraformaldehyde. The tissue was dehydrated, transparent, and embedded in paraffin, and 5 μm thick sections were made. HE staining was used to observe the morphological changes of the tissue; the immunohistochemical staining process was as follows: the sections were dehydrated to water, repaired with citrate buffer (pH 6.0) by microwave for 15 min, cooled, and washed 3 times with PBS; 3% H₂O₂ was incubated at room temperature for 10 min to eliminate endogenous peroxidase activity, washed 3 times with PBS; 5% BSA was incubated at room temperature for 1 h; the CK19 primary antibody (diluted 1:200) was incubated at 5 °C overnight, washed 3 times with PBS; the biotin-labeled secondary antibody (diluted 1:500) was incubated at room temperature for 1 h, washed 3 times with PBS; the horseradish peroxidase-labeled streptavidin (diluted 1:500) was incubated at room temperature for 30 min, washed 3 times with PBS; DAB staining was performed, and hematoxylin was counterstained, followed by dehydration, transparency, and mounting. Images were observed and collected under an optical microscope.

## Results

### General appearance and SEM microscopic morphology of PEGDA and PEGDA/DCM hydrogels

#### Appearance characterization during the photopolymerization process

**Fig. 1:**
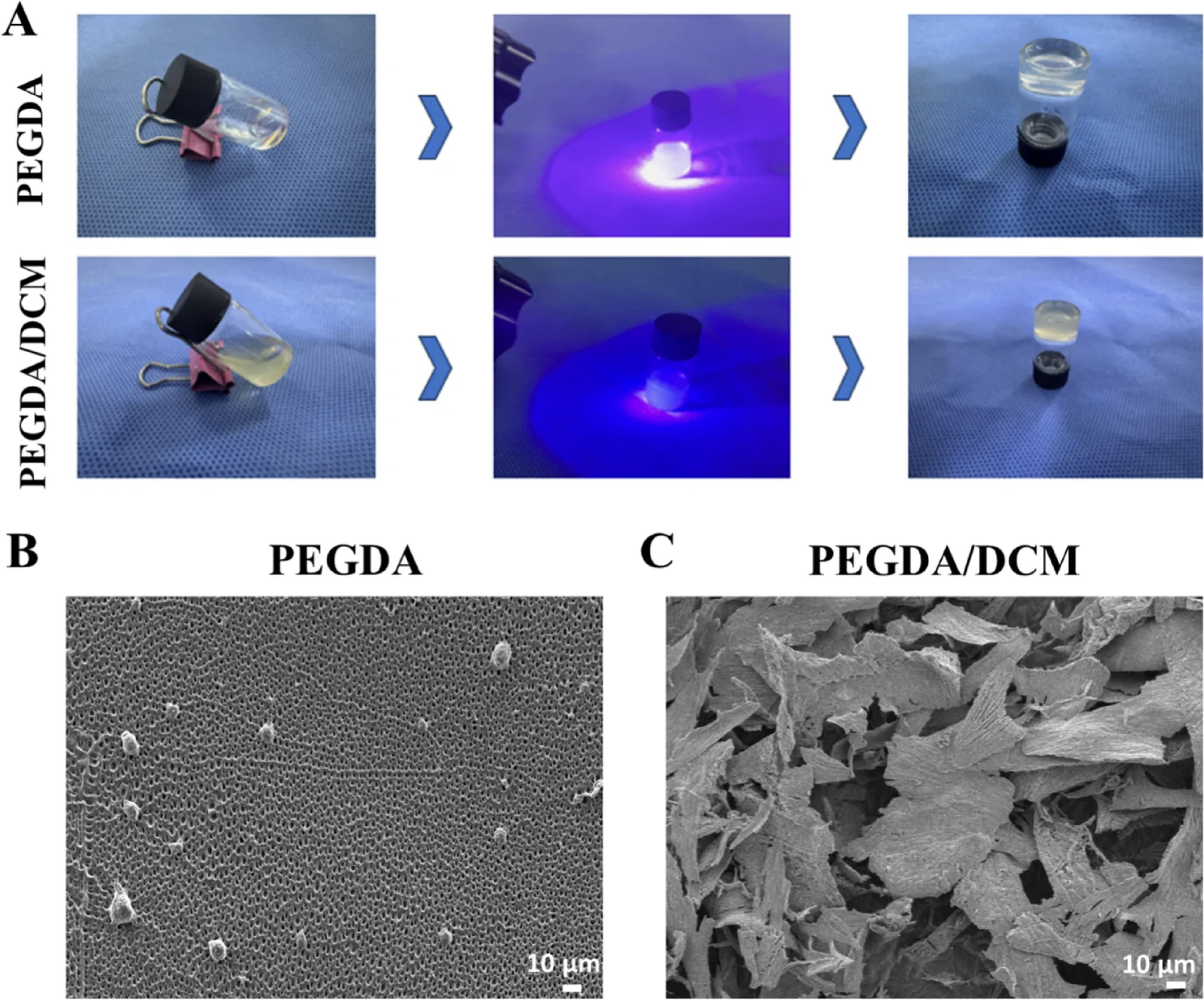
(**A**) The photopolymerization process of PEGDA and PEGDA/DCM hydrogels; (**B**) Scanning electron microscopy (SEM) images of of PEGDA hydrogel; (**C**) PEGDA/DCM hydrogel, *n* = 3 per group

The single-component PEGDA hydrogel and the PEGDA/DCM composite hydrogel were prepared by UV photopolymerization. Before crosslinking, the PEGDA pre-polymer solution was in a transparent and clear liquid state; while the PEGDA/DCM composite pre-polymer solution, due to the uniform dispersion of the cell-free extracellular matrix (DCM) powder, presented a light yellow semi-transparent liquid without obvious particle agglomeration. After 60 s of 15 mW/cm² UV irradiation, both pre-polymer solutions rapidly underwent crosslinking and solidification, forming soft and complete hydrogel blocks without leakage or uncrosslinked areas (Fig. 1A). This result indicates that the photopolymerization parameters used in this study can effectively achieve the formation of both hydrogels, and the introduction of DCM does not interfere with the photopolymerization efficiency of PEGDA, verifying the feasibility of the composite hydrogel preparation process.

#### SEM characterization of the microscopic morphology of the hydrogels

The microstructure of the hydrogels was investigated by scanning electron microscopy (SEM) using lyophilized samples. PEGDA single-component hydrogel presented a relatively regular honeycomb-like pore structure, with relatively uniform pore sizes (pore diameter distribution approximately 8–12 μm), thin pore walls and a single structure, and only a few amorphous polymer protrusions could be seen, with a lack of biomimetic features in the overall topological morphology (Fig. 1B). PEGDA/DCM composite hydrogel was significantly regulated, the original regular honeycomb-like pores were modified by the DCM-derived sheet-like and fiber-like matrix components, forming a more complex multi-level network structure. The connectivity of the pores was significantly enhanced, the pore diameter distribution expanded to 10–15 μm, and local areas could also see the natural extracellular matrix fibers retained by DCM (Fig. 1C). The above results indicate that the introduction of DCM can change the microscopic pore topology of PEGDA hydrogels, transforming them from a single uniform honeycomb structure to a more natural multi-level interconnected structure.

### Mechanical properties of PEGDA and PEGDA/ECM hydrogels

**Fig. 2:**
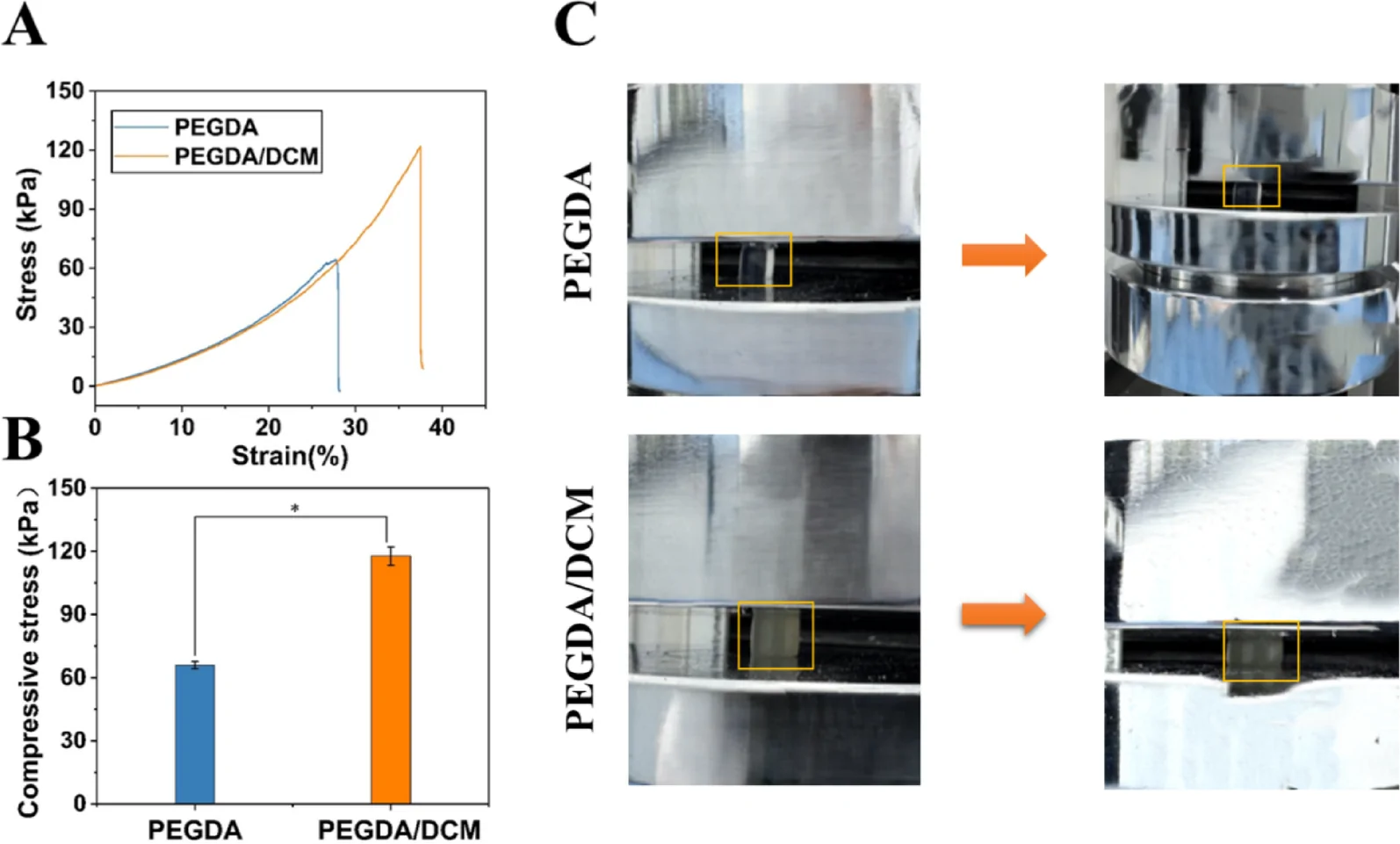
(**A**): Stress-strain curves of PEGDA and PEGDA/DCM hydrogels; (**B**): Maximum stress of PEGDA and PEGDA/DCM hydrogel; (**C**): Visual view of the deformation of PEGDA and PEGDA/DCM hydrogels during compression; * *p* < 0.05, *n* = 3 per group

Uniaxial compression tests were performed on cylindrical hydrogel samples (8 mm in diameter, 5 mm in height) using a universal testing machine. From a gross observation perspective, both groups of hydrogels exhibited a certain degree of elasticity during compression (Fig. 2C). For the pure PEGDA hydrogel, when the strain was ≤ 20%, the stress increased slowly and linearly with strain; when the strain exceeded 20%, the stress increase rate accelerated, but the stress curve fluctuated, followed by an interruption in the stress growth trend. For the PEGDA/DCM composite hydrogel, when the strain was ≤ 25%, there was no significant difference in the slope of linear stress increase with strain compared with the pure PEGDA group. After the strain exceeded 25%, the stress increased steadily with strain, and stress fluctuation did not occur until the strain reached approximately 30%. This indicated that the collapse process of internal pores in the material was more uniform, and the structural toughness was improved compared with the pure PEGDA hydrogel (Fig. 2A).

By comparing the maximum compressive stress of the two groups of hydrogels, it was found that the maximum compressive stress of the pure PEGDA hydrogel was (65.2 ± 4.8) kPa; after the incorporation of DCM, the maximum compressive stress of the PEGDA/DCM composite hydrogel significantly increased to (121.5 ± 6.3) kPa (Fig. 2B). These results confirmed the reinforcing effect of DCM on the mechanical properties of the hydrogel.

### Rheological characterization of PEGDA and PEGDA/DCM hydrogels

**Fig. 3:**
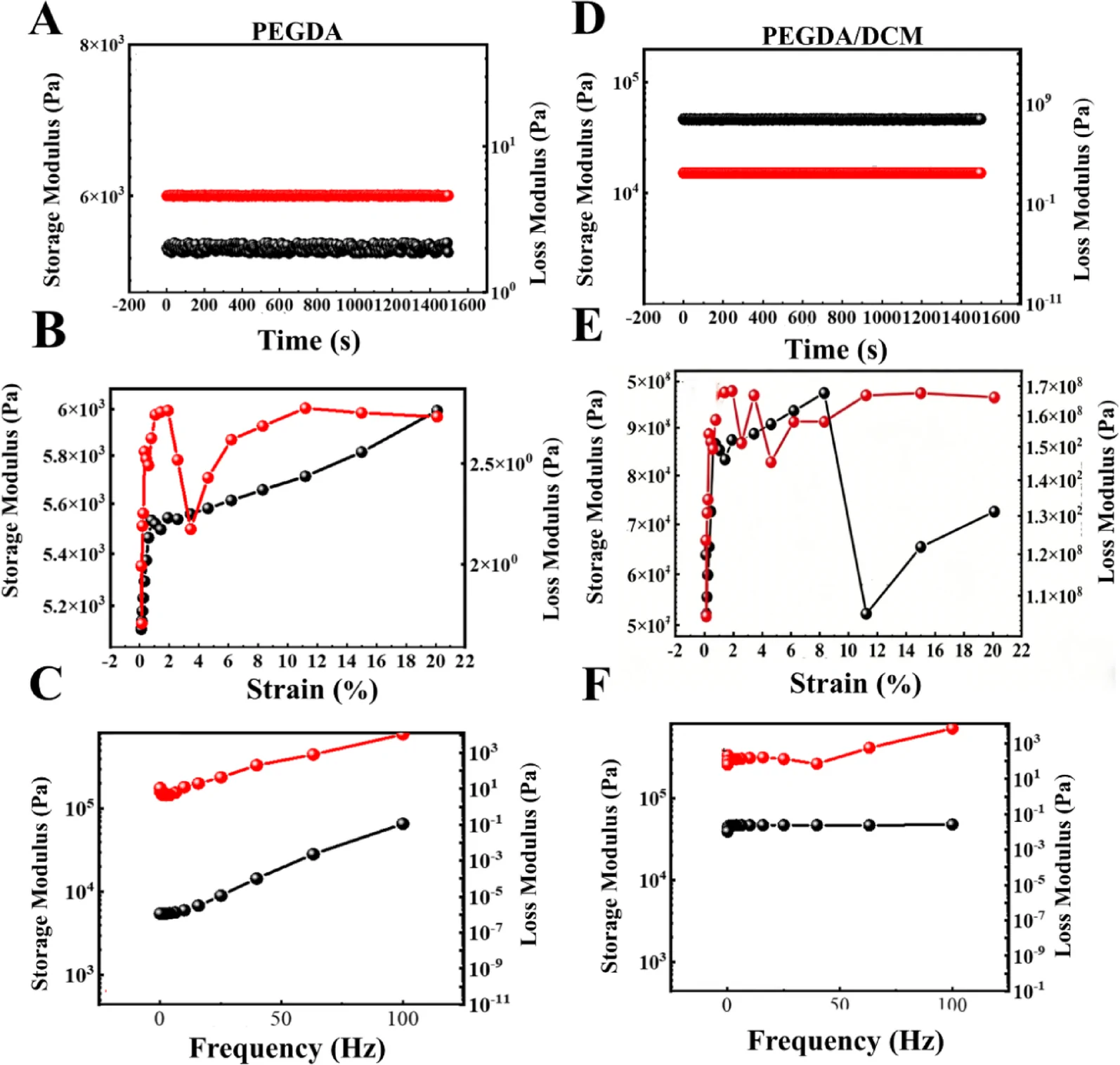
(**A**) Variations in storage modulus (G′) and loss modulus (G″) of PEGDA hydrogel over time; (**B**) Variations in storage modulus (G′) and loss modulus (G″) of PEGDA hydrogel with strain; (**C**) Variations in storage modulus (G′) and loss modulus (G″) of PEGDA hydrogel with frequency; (**D**) Variations in storage modulus (G′) and loss modulus (G″) of PEGDA/DCM hydrogel over time; (**E**) Variations in storage modulus (G′) and loss modulus (G″) of PEGDA/DCM hydrogel with strain; (**F**) Variations in storage modulus (G′) and loss modulus (G″) of PEGDA/DCM hydrogel with frequency. Black circles represent storage modulus (G′), and red circles represent loss modulus (G″). *n* = 3 per group

The dynamic viscoelastic properties of PEGDA and PEGDA/DCM hydrogels were systematically investigated via rheological measurements, including strain sweep (1 Hz, 0–20% strain), frequency sweep (1% strain, 0–100 Hz), and time sweep (1 Hz, 0–1500 s), to characterize their elastic-viscous balance and structural stability. For the time sweep response, both PEGDA and PEGDA/DCM hydrogels exhibited outstanding long-term mechanical stability within 0–1500 s, with stable plateaus for storage modulus (G′) and loss modulus (G″); notably, the moduli of PEGDA/DCM were approximately two orders of magnitude higher than those of pure PEGDA, demonstrating that the incorporation of DCM significantly enhanced the cross-linking density and mechanical strength while maintaining structural stability (Fig. 3A and B). In the strain sweep tests, pure PEGDA hydrogel possessed a narrow linear viscoelastic region (LVR) at strain < 2%, and its G′ and G″ decreased initially and then increased with strain hardening behavior beyond the LVR, whereas PEGDA/DCM hydrogel exhibited a remarkably wider LVR (strain < 8%), followed by a sharp drop and subsequent recovery of G′ and G″ upon further increasing strain (Fig. 3C and D). Regarding frequency sweep measurements, G′ and G″ of PEGDA hydrogel increased with elevated frequency (with G′ > G″) and showed an accelerated growth rate above 50 Hz, indicating frequency-dependent viscoelasticity. In contrast, PEGDA/DCM hydrogel displayed a nearly constant *G*′ plateau (on the order of 10⁵ kPa) and frequency-independent G″ (much lower than G′) across 0–100 Hz, revealing superior dynamic structural stability against varying loading rates (Fig. 3E, 3 F).

### Swelling kinetics curves of PEGDA and PEGDA/DCM hydrogels

**Fig. 4:**
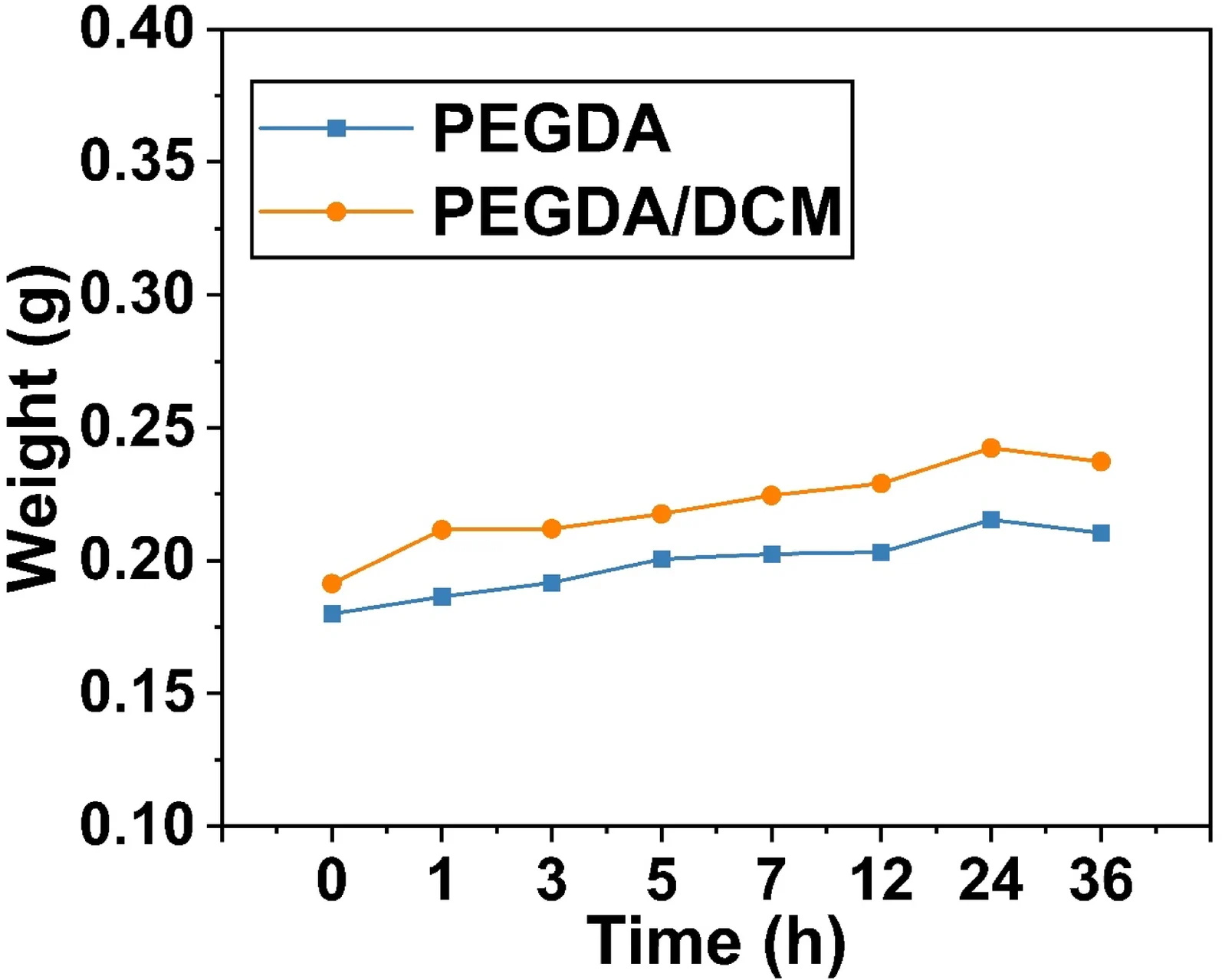
Swelling kinetic curves of PEGDA and PEGDA/DCM hydrogels, *n* = 3 per group

Swelling experiments were performed to characterize the water absorption and swelling behavior of hydrogels in a simulated physiological environment (37 °C PBS solution). Both groups of hydrogels exhibited a typical kinetic characteristic of “rapid swelling-slow equilibrium”: In the rapid swelling stage (0–12 h), the weight of both hydrogels continued to increase with prolonged immersion time, indicating that the aqueous medium rapidly penetrated into the porous network of hydrogels through diffusion; among which the weight growth rate of PEGDA/DCM hydrogel was slightly higher than that of pure PEGDA hydrogel. In the slow equilibrium stage (12–36 h), the swelling rate of both hydrogels slowed down significantly after 12 h, and the weight change tended to level off, gradually approaching the swelling equilibrium state. By 36 h, the swelling weight of PEGDA stabilized at approximately 0.21 g, while that of PEGDA/DCM stabilized at around 0.24 g (Fig. 4). The swelling behavior of hydrogels directly affects their physicochemical properties and biocompatibility in vivo. Excessive swelling can induce tracheal lumen stenosis and impair ventilation, whereas appropriate swelling capacity enables hydrogels to adapt to the in vivo humoral environment and maintain the structural stability of their three-dimensional network.

### In vitro cumulative release curve of EGF from PEGDA/DCM composite hydrogels

**Fig. 5:**
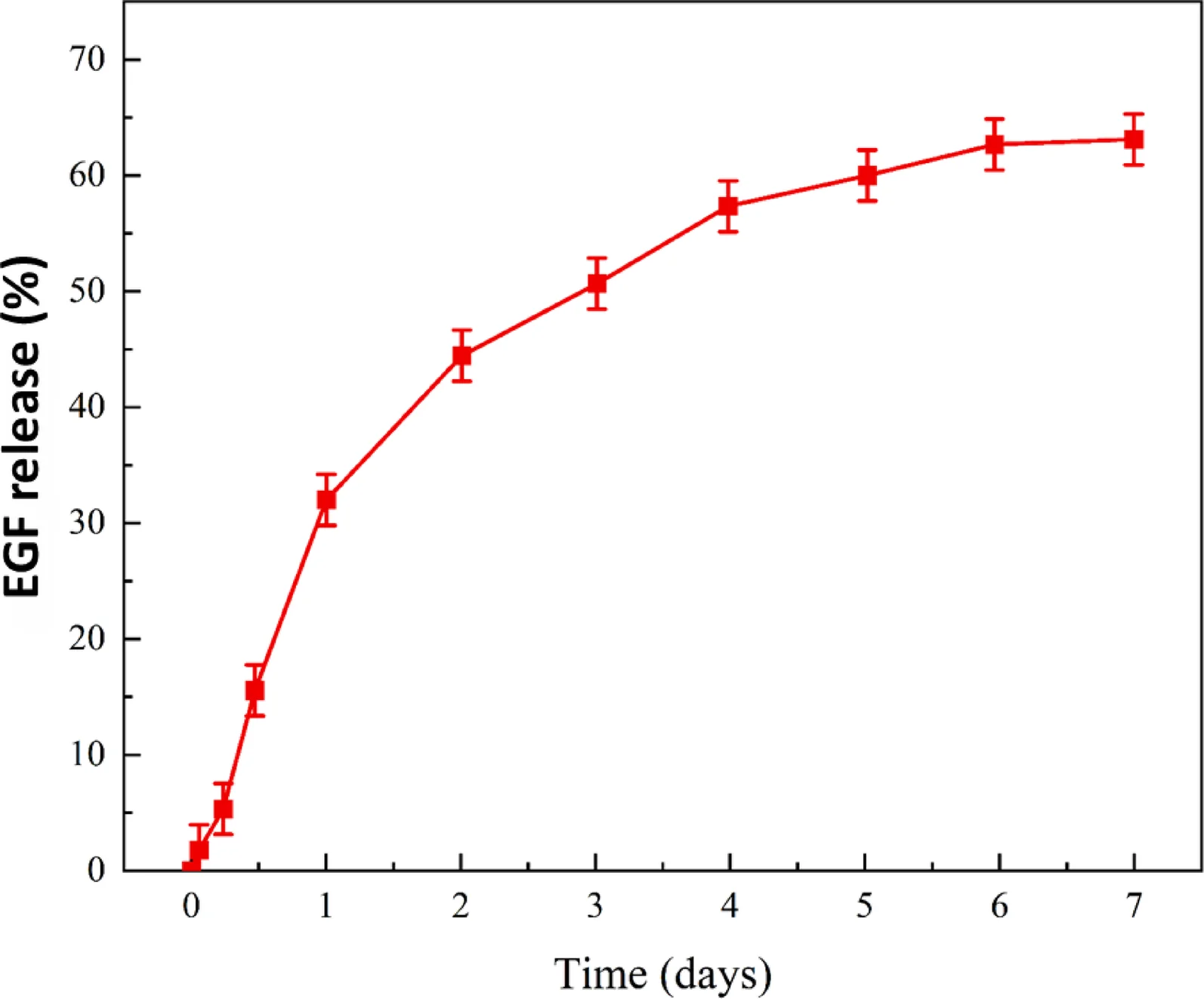
In vitro cumulative release curve of EGF from PEGDA/DCM composite hydrogels, *n* = 3 per group

In this section, the EGF release behavior of EGF-loaded PEGDA/DCM composite hydrogels in PBS solution at 37 °C was determined using a standard ELISA assay. Within 0–1 day, the cumulative EGF release rate increased rapidly to approximately 30%, representing the fastest release phase. This might be attributed to the rapid diffusion of loosely bound EGF on the hydrogel surface into the external environment. From day 1 to day 6, the release rate slowed down gradually, with the cumulative release rate rising steadily; by day 6, the cumulative release rate reached around 63%. Thereafter, the release tended to stabilize, maintaining at approximately 63% on day 7 without obvious termination or sudden increase (Fig. 5).

### In vitro biocompatibility evaluation results of composite hydrogels

#### Live/dead staining results of tracheal epithelial cells

**Fig. 6:**
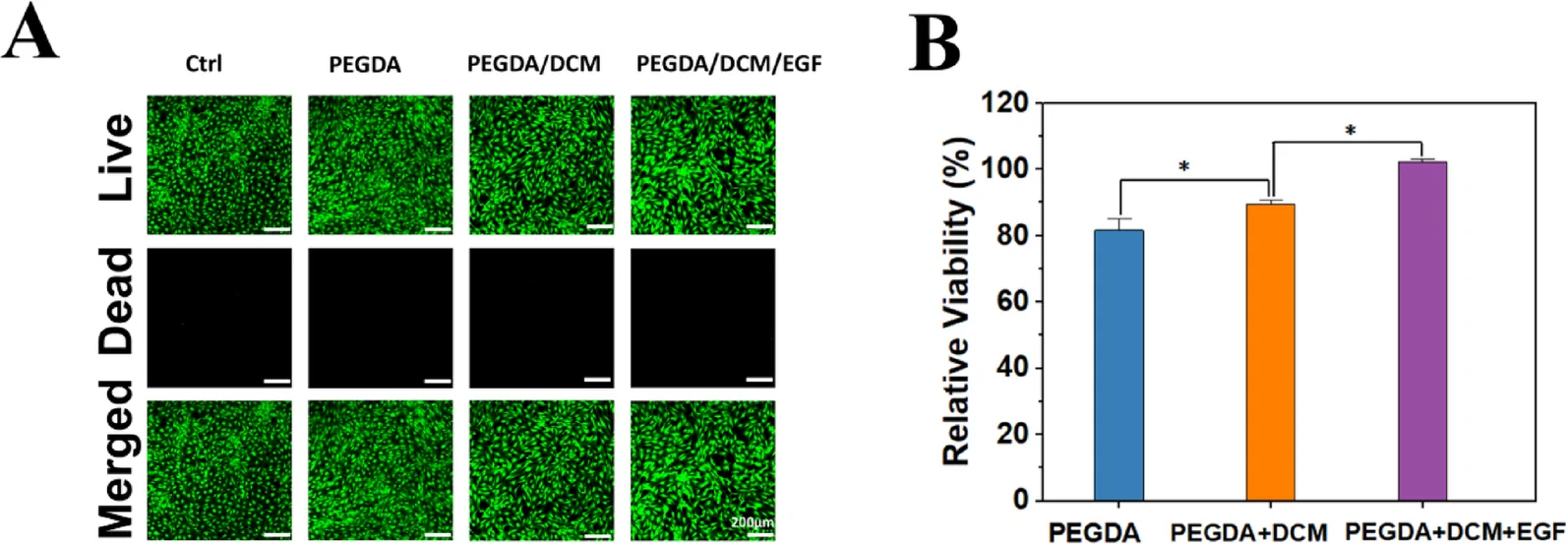
(**A**) Live/dead staining images of tracheal epithelial cells cultured with extracts from PEGDA, PEGDA/DCM, and PEGDA/DCM/EGF hydrogel ; scale bar = 200 μm; (**B**) Relative survival rate of tracheal epithelial cells cultured with extracts from PEGDA, PEGDA/DCM, and PEGDA/DCM/EGF hydrogel (*p < 0.05)

Live/dead staining assay was employed to evaluate the viability of rat tracheal epithelial cells cultured with extracts of different hydrogels for 3 days. In the green (live-cell) channel, viable cells in the control group, PEGDA group, PEGDA/DCM group and PEGDA/DCM/EGF group all exhibited intense green fluorescence and presented the typical polygonal morphology of epithelial cells. In the red (dead-cell) channel, no obvious red fluorescence was detected in all experimental and control groups, with only occasional scattered red signals observed, indicating that none of the hydrogels induced significant cytotoxicity and the cell mortality rate was extremely low. In the merged images, green fluorescence was absolutely predominant in all groups, which further verified the favorable cytocompatibility of each hydrogel (Fig. 6A).

#### Relative Survival Rate of Tracheal Epithelial Cells (CCK-8 Assay)

The CCK-8 assay was conducted to detect cell proliferative activity. The relative viability of cells in the PEGDA extract group was (81.2 ± 3.5) %, which was the lowest among the three groups, indicating that the pure PEGDA hydrogel extract had no obvious cytotoxicity but exhibited weak proliferation-promoting activity. After the incorporation of DCM, the relative viability of cells in the PEGDA/DCM extract group was significantly increased to (90.5 ± 4.1) %, suggesting that the introduction of DCM could enhance the bioactivity of the extract and facilitate cell proliferation. With the further addition of EGF, the relative viability of the PEGDA/DCM/EGF extract group was further elevated to (102.3 ± 2.8) % (Fig. 6B).

### Scratch wound healing assay results of tracheal epithelial

**Fig. 7:**
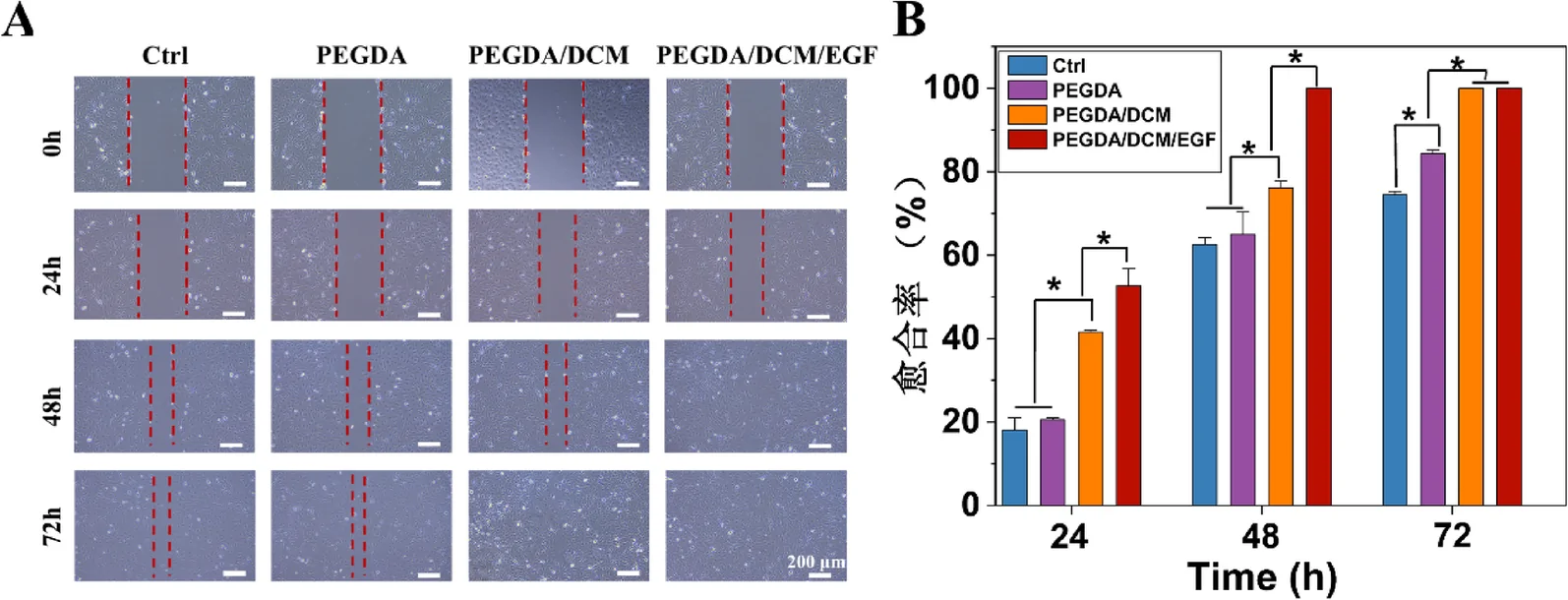
(**A**) Scratch morphology images observed at different time points; (**B**) Quantitative statistical graph of scratch healing rates at the corresponding time points; *: *p* < 0.05, red dashed lines mark the initial scratch boundaries, scale bar = 200 μm

The scratch wound healing assay was performed to evaluate the effect of different hydrogel extracts on the migration ability of rat tracheal epithelial cells. At the initial time point (0 h), the scratch width was consistent across all experimental groups (PEGDA, PEGDA/DCM, PEGDA/DCM/EGF) and the control group (Ctrl), with uniform cell-free areas clearly marked by red dashed lines to ensure consistent initial experimental conditions, and the healing rate was 0% in all groups. After 24 h of culture, the scratch width in the Ctrl group contracted slightly with a still wide cell-free area and a healing rate of approximately 20%, while the PEGDA extract group showed a similarly wide cell-free area and a healing rate of around 20%; in contrast, the scratch width in the PEGDA/DCM extract group was significantly reduced, with the cell-free area accounting for approximately 45% of the initial size and a healing rate of about 55%, and the PEGDA/DCM/EGF extract group exhibited the most obvious scratch contraction, with the cell-free area less than 40% of the initial width and a healing rate of roughly 55%. By 48 h, the scratch in the Ctrl group contracted further but a clear cell-free strip remained, with a healing rate of 62%; the PEGDA group showed slow scratch healing with a still wide cell-free area and a healing rate of approximately 65%; the PEGDA/DCM group displayed a substantial reduction in scratch width, leaving only a narrow cell-free area and a healing rate of 80%; and the PEGDA/DCM/EGF group showed nearly closed scratches, with the cell-free area basically covered by cells and a healing rate of 100%. At 72 h post-scratching, the scratch in the Ctrl group remained unclosed with residual cell-free areas and a healing rate of approximately 75%; the PEGDA group still had partially unclosed scratches with a healing rate of about 85%; the PEGDA/DCM group achieved complete scratch closure with a healing rate of 100%; and the PEGDA/DCM/EGF group not only showed fully closed scratches but also formed a continuous monolayer of spread cells in the original scratch area, maintaining a healing rate of 100%.The migration ability of tracheal epithelial cells is one of the core links in tracheal defect repair (cells need to migrate to cover the defect area to reconstruct a continuous epithelial layer) (Fig. 7A and B).

These results demonstrate that the PEGDA/DCM/EGF composite hydrogel extract can effectively enhance cell migration ability, laying a cellular functional foundation for the repair of tracheal epithelial defects in vivo.

### Repair of rat tracheal defect model

#### In vivo status of scaffolds and gross characteristics of defect healing

**Fig. 8:**
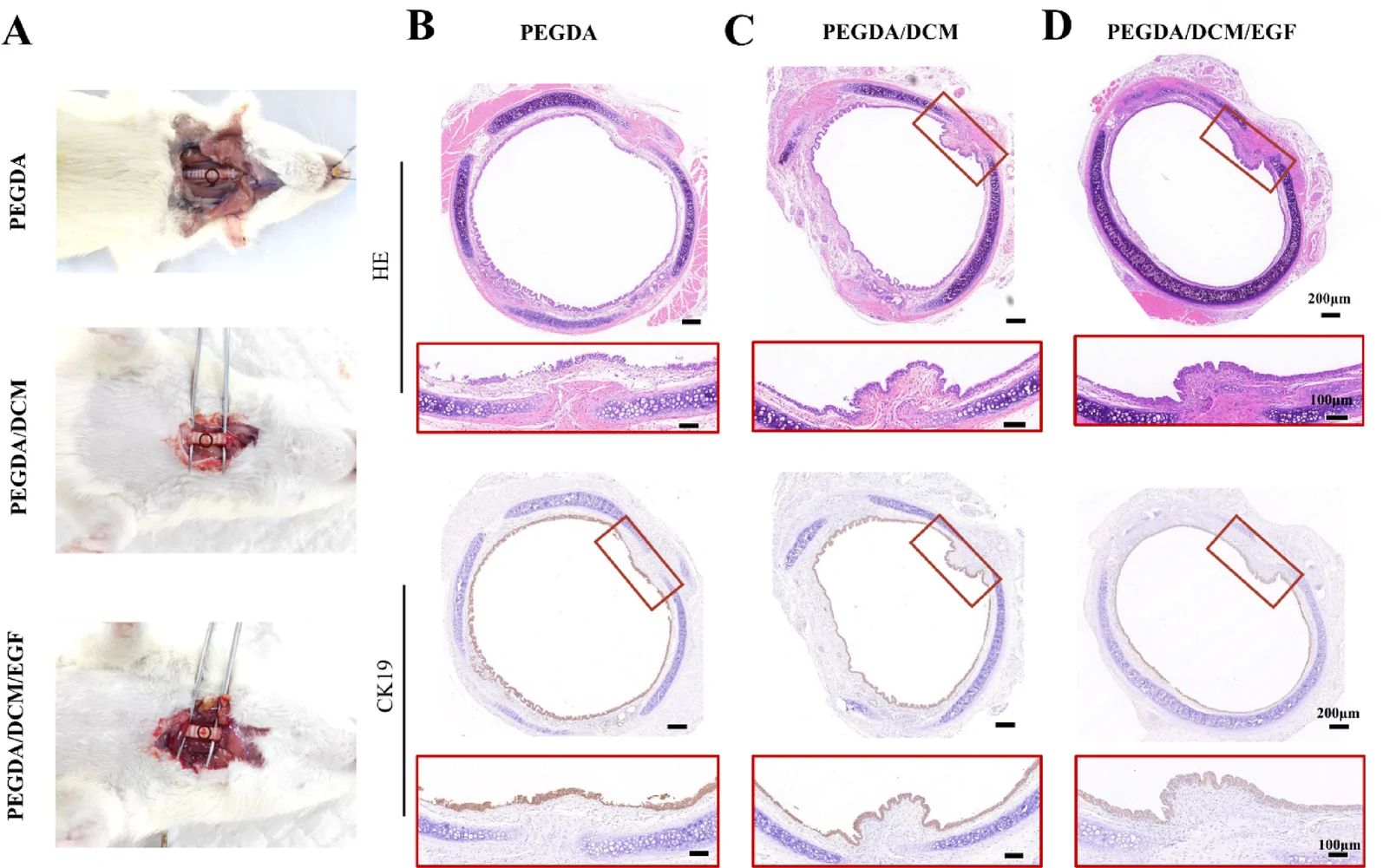
(**A**) Gross images of harvested samples at 2 weeks post-implantation of PEGDA, PEGDA/DCM, and PEGDA/DCM/EGF hydrogels; (**B**) HE staining and CK19 staining of the repair site in the PEGDA group; (**C**) PEGDA/DCM group; (**D**) PEGDA/DCM/EGF group

We present the gross sample outcomes of 2-mm tracheal defect models in Sprague-Dawley (SD) rats at 2 weeks post-implantation with different hydrogels. After tracheal exposure, no rupture was observed at the repair site in any of the three hydrogel groups. In the pure PEGDA hydrogel group, the hydrogel exhibited poor integration with the surrounding tracheal tissue, with fibrous adhesion visible at the defect edges and mild inflammatory exudates observed locally. In contrast, the PEGDA/DCM and PEGDA/DCM/EGF composite hydrogel groups showed more complete tissue coverage over the tracheal defects, with blurred boundaries at the defect sites. These results demonstrated that the incorporation of DCM effectively enhanced the in vivo tissue affinity and in-situ stability of the scaffolds (Fig. 8A).

#### Histological characteristics of HE-stained sections in each group

Hematoxylin and eosin (H&E) staining revealed that the defect area in the pure PEGDA hydrogel group presented incomplete epithelial coverage, with disordered arrangement of epithelial cells that failed to form the normal pseudostratified ciliated columnar epithelium. In the PEGDA/DCM composite hydrogel group, by contrast, the epithelial layer showed markedly improved continuity, with only local, mild irregularities in cell arrangement and preliminary formation of a pseudostratified structure. Compared with the PEGDA group, the PEGDA/DCM group achieved superior healing at the defect edges, as evidenced by more complete epithelial coverage and a better-organized epithelial lining, indicating enhanced epithelial regeneration capacity. Notably, the PEGDA/DCM/EGF composite hydrogel group displayed a continuous and intact epithelial layer, where epithelial cells were arranged in a typical pseudostratified ciliated columnar pattern with regular morphology and uniform density; compared with the PEGDA group, the PEGDA/DCM/EGF group showed the most favorable epithelial healing, with fully regenerated and properly organized pseudostratified ciliated columnar epithelium covering the entire defect site (Fig. 8B–D).

#### Characteristics of cytokeratin 19 (CK19) positive expression in samples from each group

In this study, CK19 immunohistochemical (IHC) staining (positive expression is indicated by brown staining) was performed to evaluate the regeneration and integrity of the tracheal epithelial layer after repair with different hydrogel scaffolds. Under high magnification, the pure PEGDA hydrogel group showed sparse and discontinuous CK19-positive staining (brown) areas, with only a small number of scattered positive cells locally. The epithelial layer structure was disrupted, lacking complete epithelial coverage, indicating that the pure PEGDA scaffold could not fully and effectively induce the regeneration and colonization of tracheal epithelial cells. In contrast, the PEGDA/DCM composite hydrogel group exhibited significantly expanded CK19-positive staining areas and increased positive cell density. The epithelial layer showed continuity but failed to fully develop into a pseudostratified epithelial structure, demonstrating that the introduction of DCM can promote the regeneration of tracheal epithelial cells, while the integrity of the epithelial layer still needs optimization. Notably, under high magnification, the PEGDA/DCM/EGF composite hydrogel group displayed continuous and intact CK19-positive staining areas, with uniform brown positive signals covering the entire luminal epithelial layer. Positive cells were closely arranged with regular morphology, which was highly consistent with the CK19 expression pattern of normal tracheal epithelium (Fig. 8B-D). These results indicated that the incorporation of EGF into the PEGDA/DCM/EGF composite hydrogel optimizes its ability to promote the growth and migration of epithelial cells.

## Discussion

The core challenge in tracheal defect repair is to simultaneously satisfy three critical requirements: stable mechanical support, favorable biocompatibility, and potent tissue regeneration inductivity. In this study, poly (ethylene glycol) diacrylate (PEGDA) was selected as the hydrogel matrix due to its photo-crosslinking property that enables rapid gelation and customizable shaping. Its three-dimensional network can mimic the physical microenvironment of the extracellular matrix (ECM). However, the inherent limitation of insufficient cell affinity of pure PEGDA was effectively mitigated by the introduction of decellularized tracheal matrix (DCM). As a natural tissue-derived material, DCM retains core components including collagen, elastin, and glycosaminoglycans (GAGs). These biomacromolecules not only provide integrin-mediated adhesion sites for cells (e.g., specific binding between fibronectin and α5β1 integrin) but also regulate the pore topology of the hydrogel via their native fibrous architecture [28], transforming the single honeycomb-like pore structure of pure PEGDA into a hierarchically interconnected bionic structure that facilitates nutrient transport and cell migration.The loading and sustained release of epidermal growth factor (EGF) further enhanced the bioactivity of the scaffold [29–32]. This ternary composite design integrating “PEGDA-based mechanical support, DCM-derived bionic microenvironment, and EGF-mediated signal regulation” achieves synergy between physical properties and biological functions, providing a comprehensive solution for tracheal defect repair.

As a dynamic respiratory organ, the trachea must maintain lumen patency under cyclic mechanical loading, with a physiological compressive stress range of approximately 80–150 kPa. The maximum compressive stress of the PEGDA/DCM/EGF composite hydrogel was (121.5 ± 6.3) kPa, which perfectly falls within this physiological range. Its stable stress response during strain hardening and excellent deformation resistance effectively prevent excessive deformation or collapse during respiratory movements. Rheological tests demonstrated that the composite scaffold exhibited superior storage modulus (G’) and linear viscoelasticity compared with the pure PEGDA scaffold. This mechanical optimization is attributed to the interpenetrating network formed by DCM fibers and PEGDA polymer chains, which enhances deformation capacity and structural toughness through stress dispersion.

Swelling tests demonstrated that neither the PEGDA/DCM hydrogel nor the pure PEGDA hydrogel exhibited obvious water absorption and swelling. The PEGDA/DCM hydrogel presented a relatively higher equilibrium swelling capacity than the pure PEGDA group. Appropriate swelling capacity enabled the hydrogel to rapidly adapt to the in vivo humoral environment, maintain the stability of its three-dimensional network, and provide sufficient space for cell adhesion, proliferation and substance exchange. Meanwhile, the PEGDA/DCM group avoided mechanical property attenuation and tracheal lumen stenosis induced by excessive swelling.

Live/dead staining and CCK-8 assay confirmed that both the PEGDA/DCM/EGF and PEGDA/DCM hydrogels had a stronger ability to promote the proliferation of tracheal epithelial cells compared with the pure PEGDA hydrogel. Tracheal epithelial cells cultured with the PEGDA/DCM/EGF hydrogel showed the highest survival rate and proliferative activity, which was attributed to the synergistic regulation of DCM and EGF. Studies have confirmed that collagen in DCM activates the FAK-Src signaling pathway through integrin αvβ3, promoting the formation of focal adhesions and cytoskeleton rearrangement [33, 34], thus providing a physical anchorage and signal transduction basis for cell proliferation. EGF binds to cell surface receptors, activates the PI3K-Akt and MAPK/ERK signaling pathways, upregulates the expression of proliferation-related genes, and accelerates the progression of the cell cycle [35, 36].Their synergy provides sufficient adhesion support and proliferation signals for cells in the bionic microenvironment. Scratch wound healing assays showed that the PEGDA/DCM/EGF group exhibited significantly faster healing rate than the other groups, achieving complete closure with continuous cell layer formation at 72 h, which meets the core demand of tracheal epithelial defect repair.

Gross in vivo observation revealed that the PEGDA/DCM/EGF scaffold maintained a stable position within 2 weeks after implantation, closely adhering to the host tracheal tissue without displacement, detachment, or obvious inflammation. In contrast, the pure PEGDA scaffold exhibited poor tissue integration, with obvious fibrous adhesion forming at the edges. This difference is mainly attributed to the native ECM components preserved in DCM, which effectively facilitate host cell infiltration into the scaffold. Meanwhile, the low immunogenicity of DCM further helps reduce adverse host immune rejection at the implantation site [24, 37].

Histological analysis yielded consistent findings. In the PEGDA/DCM/EGF group, the regenerated epithelium remained continuous and intact, with negligible inflammatory infiltration within the submucosal tissue. The improved repair performance benefited from the ternary composite design. This design integrated structural support, biomimetic microenvironment modulation, and biological signal induction, whereas conventional scaffolds typically focus on a single functional dimension. As a specific biomarker for airway epithelial cells, CK19 was detected with continuous and uniform positive expression across the luminal surface in the PEGDA/DCM/EGF group, confirming complete epithelial coverage at the defective site.

However, the full physiological function of regenerated epithelial cells remains to be explored. EGF has been shown to upregulate MUC5 gene expression, promoting mucous phenotypic differentiation of tracheal epithelial cells while maintaining epithelial polarity and barrier function—critical for preventing airway dryness and resisting pathogen invasion [38]. Subsequent studies will require additional staining for cilia, mucins, and specific functional cell markers to validate the differentiation degree and functional maturation of regenerated epithelium.

Notably, the PEGDA/DCM and PEGDA/DCM/EGF groups exhibited mild luminal tissue ingrowth at the early remodeling stage, which was absent in the pure PEGDA group. Histologically, this ingrown tissue stained positive for CK19, confirming the presence of regenerative airway epithelial cells rather than uncontrolled pathological fibrosis. This phenomenon likely arises from the enhanced mechanical stability and prolonged degradation kinetics of DCM-modified hydrogels, which provide sustained structural support and pro-regenerative cues to promote cell infiltration and tissue remodeling at the defect site. While this epithelialized tissue ingrowth may contribute to re-epithelialization of the tracheal mucosa during early repair, the long-term risk of excessive granulation tissue formation and luminal stenosis cannot be overlooked. Therefore, further long-term in vivo studies are currently underway to systematically evaluate tracheal patency, tissue maturation, and potential complications over an extended period, to validate the safety and efficacy of these hydrogel scaffolds for clinical translation.

The PEGDA/DCM/EGF composite hydrogel scaffold exhibits excellent comprehensive performance in tracheal defect repair but has several limitations. First, the 2-week observation period is insufficient; long-term (≥ 3 months) scaffold degradation, tissue regeneration stability, and epithelial function recovery require further verification. While CK19 staining confirms epithelial regeneration and continuous coverage, the terminal differentiation (e.g., cilia formation, mucin secretion) and complete physiological function of regenerated epithelial cells remain unvalidated—critical for maintaining long-term tracheal patency and functional integrity. Second, angiogenesis in the repair area has not been systematically evaluated, despite its key role in ensuring long-term tissue survival. Third, the study only used a rat model, and differences in tracheal anatomy and mechanical environment between rats and humans necessitate verification in large animal models (e.g., dogs, pigs) for clinical translation.

Future research will focus on several aspects: (1) optimizing scaffold degradation rate by adjusting PEGDA crosslinking degree and DCM content to match scaffold degradation with tissue regeneration; (2) introducing pro-angiogenic factors (e.g., VEGF) to construct a multi-factor synergistic sustained-release system for accelerating blood supply reconstruction; (3) combining 3D bioprinting for personalized scaffold customization to adapt to diverse clinical defect sizes and anatomical forms; (4) conducting long-term large animal experiments to evaluate biosafety and clinical applicability, providing experimental basis for clinical translation; (5) performing specific staining for cilia, mucins, and ionocytes combined with functional detection to verify epithelial terminal differentiation and functional maturation, clarify EGF’s regulatory role in tracheal epithelial differentiation, and further improve repair efficacy.

## Conclusion

The PEGDA/DCM/EGF composite hydrogel fabricated in this study exhibits favorable moldability. It can be custom shaped to match the actual size and morphology of tracheal defects in thoracic surgery, showing good adaptability to relevant clinical demands. Meanwhile, the composite hydrogel possesses satisfactory biocompatibility and enables sustained mild release of EGF, which effectively promotes epithelial regeneration at the defect site. Overall, the material meets the basic safety and efficacy requirements for thoracic surgical repair scaffolds, providing a promising experimental candidate for further development in clinical tracheal defect reconstruction.

## Data Availability

The data that support the findings of this study are available from the corresponding author upon reasonable request. All raw data related to material characterization, in vitro cell experiments, and in vivo tracheal defect repair studies are stored in the laboratory of the corresponding author (Qingxu Hao) at Soochow University and can be accessed for research purposes with appropriate permission.

## Abbreviations

PEGDA: Polyethylene glycol diacrylate
DCM: Decellularized matrix
EGF: Epidermal growth factor
RTEpiC: Rat tracheal epithelial cells
SD: Sprague-Dawley
HE: Hematoxylin-eosin
SEM: Scanning electron microscopy
ELISA: Enzyme-linked immunosorbent assay
G’: Storage modulus
G’’: Loss modulus
CCK-8: Cell Counting Kit-8
CK19: Cytokeratin 19
OD: Optical density
